# Does mpMRI guidance improve HIFU partial gland ablation compared to conventional ultrasound guidance? Early functional outcomes and complications from a single center

**DOI:** 10.1590/S1677-5538.IBJU.2019.0682

**Published:** 2020-09-02

**Authors:** Arjun Sivaraman, Giancarlo Marra, Armando Stabile, Annick Mombet, Petr Macek, Camille Lanz, Nathalie Cathala, Marco Moschini, Arie Carneiro, Rafael Sanchez-Salas, Xavier Cathelineau

**Affiliations:** 1 Department of Urology L’Institut Mutualiste Montsouris Université Paris Descartes Paris France Department of Urology, L’Institut Mutualiste Montsouris, Université Paris Descartes, Paris, France;; 2 Department of Urology San Giovanni Battista Hospital Città della Salute e della Scienza and University of Turin Turin Italy Department of Urology, San Giovanni Battista Hospital, Città della Salute e della Scienza and University of Turin, Turin, Italy

**Keywords:** Prostate cancer, familial [Supplementary Concept], High-Intensity Focused Ultrasound Ablation, complications [Subheading]

## Abstract

**Background:**

Focal therapy (FT) for localized prostate cancer (PCa) treatment is raising interest. New technological mpMRI-US guided FT devices have never been compared with the previous generation of ultrasound-only guided devices.

**Materials and Methods:**

We retrospectively analyzed prospectively recorded data of men undergoing FT for localized low- or intermediate-risk PCa with US- (Ablatherm®-2009 to 2014) or mpMRI-US (Focal One®-from 2014) guided HIFU. Follow-up visits and data were collected using internationally validated questionnaires at 1, 2, 3, 6 and 12 months.

**Results:**

We included n=88 US-guided FT HIFU and n=52 mpMRI-US guided FT HIFU respectively. No major baseline differences were present except higher rates of Gleason 3+4 for the mpMRI-US group. No major differences were present in hospital stay (p=0.1), catheterization time (p=0.5) and complications (p=0.2) although these tended to be lower in the mpMRI-US group (6.8% versus 13.2% US FT group). At 3 months mpMRI-US guided HIFU had significantly lower urine leak (5.1% vs. 15.9%, p=0.04) and a lower drop in IIEF scores (2 vs. 4.2, p=0.07). Of those undergoing 12-months control biopsy in the mpMRI-US-guided HIFU group, 26% had residual cancer in the treated lobe.

**Conclusion:**

HIFU FT guided by MRI-US fusion may allow improved functional outcomes and fewer complications compared to US- guided HIFU FT alone. Further analysis is needed to confirm benefits of mpMRI implementation at a longer follow-up and on a larger cohort of patients.

## INTRODUCTION

Focal therapy (FT) for localized prostate cancer (PCa) has been introduced in the clinical scenario with the aim of reducing treatment-related drawbacks such as incontinence, erectile dysfunction and other related complications whilst preserving the oncological benefits of whole-gland treatments ( 1 , 2 ).

Interests in FT are high with two recent surveys proving the Urological community seems overall in favor of its adoption also outside an academic setting ( 3 , 4 ).

Evidence is also rapidly growing. In 2016, a systematic review already summarized the findings of more than 3.000 patients being treated, yielding optimal results in terms of continence and erectile function preservation, minimal complications and acceptable short-to-medium term oncological outcomes ( 1 ).

Nonetheless, current guidelines recommend using FT as an experimental treatment modality, hence in clinical trials only ( 5 ). Main reasons include: PCa biology and pathology, as PCas are often multifocal; imaging and biopsy limitations in patients selection, as inaccuracy may result in undertreatment, thus potentially in disease progression; absence of level 1 evidence, as no randomized controlled trials against radical treatment have been published to date ( 2 , 5 ).

Recently, PCa diagnostic pathway has been revolutionized by the use of mpMRI ( 6 , 7 ). MpMRI is not perfect yet, as it may still miss some clinically significant disease, especially when performed outside experienced centers. Nonetheless, it brings important advantages. On the one hand, mpMRI allows proven benefits in terms of PCa detection when targeting suspicious areas instead of blindly sampling the gland. On the other hand, precise identification of the target ‘’index’’ cancer focus in terms of volume and site within the prostate is also an ideal feature to guide and, thus, theoretically to enhance precision in prostate-sparing treatments ( 8 ).

As per systematic prostate biopsies, HIFU focal therapy has been initially performed relying on transrectal ultrasound only (US guided HIFU). Currently, new ‘’fusion’’ software allow to synchronize and superimpose mpMRI and ultrasound, with a view to enhancing treatment precision both in terms of treated target areas, by increasing oncological efficacy, and in terms of spared untreated tissue, by increasing functional results and decreasing complications. We hypothesized higher treatment precision deriving from mpMRI fusion-software may positively impact HIFU focal therapy outcomes.

Hence, we evaluated early functional outcomes and complications of partial gland ablation of PCa with mpMRI-US fusion guided HIFU and we compared these results with a cohort of men receiving standard US-guided HIFU.

## MATERIALS AND METHODS

### Population and inclusion criteria

We retrospectively reviewed a prospectively maintained database of men undergoing FT for localized intermediate- and low-risk PCa at Institut Mutualiste Montsouris, Paris, France. The inclusion criteria for PGA were: clinical stage T1c-T2a, maximum 33% of biopsy cores involved by PCa, Gleason ≤7 (3+4), PSA <15ng/mL, absence of extra-prostatic extension and seminal vesicle invasion and pelvic lymph node involvement at mpMRI, and patient’s life expectancy higher than 10 years. Men with anterior and/or apical lesions or men with prostate calcifications and/or cysts possibly interfering with optimal HIFU energy delivery were excluded ( 9 , 10 ).

Cancer diagnosis and localization was achieved with a combination of biopsy and imaging. All patients planned for partial gland ablation (PGA) underwent 1.5T mpMRI without endorectal coil. The mpMRI was reported by a single radiologist experienced in genital-urinary imaging. The mpMRI report included presence or absence of suspicious lesion, sectoral location of the lesion within the prostate and the PIRADS V1 grading of the lesion. The radiologist also marked the borders of the lesion with a 7-9mm safety margin. The patients underwent standard 12-cores TRUS biopsy plus mpMRI targeted fusion biopsy in case of mpMRI suspicious (PIRADS V1 >2) areas. Patients with a negative mpMRI underwent transperineal mapping biopsy (TPMB) to rule out the presence of PCa before the treatment. All men completed standard Genito-urinary functions questionnaires: International Prostate Symptom Score (IPSS), International Continence Society (ICS), and International Index of Erectile Function (IIEF-5). The Martin-Donat criteria were followed to report the surgical complications, and severity of complications was recorded using the Clavien classification.

### Treatment characteristics

#### General features

We employed US and mpMRI-US fusion guidance for HIFU from April 2009 to June 2014 and since June 2014 respectively to perform PGA (lesion ablation/hemiablation/sub-total). Both treatments were performed under general anesthesia. After treatment, a bladder catheter was placed. Patients with prostate volumes >40cc or with symptoms of lower urinary tract (IPSS>8) underwent a transurethral resection of the prostate (TURP) one month before or during the HIFU treatment. For both US and mpMRI-US procedures, the transducer was inserted into the rectum with the patient in right lateral position. PGA was obtained treating the region of interest (ROI) by keeping a safety margin of 4-6mm from the sphincter to prevent damage. All procedures were performed by two experienced surgeons (>100 partial gland HIFU ablations before the study initiation). All men provided written informed consent and the study was approved by local IRB ( 11 - 13 ).

## US-MRI guided partial ablation

Energy delivery was performed using the Focal-One® device (EDAP TMS, Vaulx-en-Velin, France) using a 3MHz transducer for the treatment combined with a 7.5MHz image transducer. During the firing phase, the software automatically controlled the position of the endorectal probe, and the cooling system maintained the rectal mucosa temperature at 14°C. The focal point position inside the prostate was controlled in real time by the surgeon.

Treatment planning was guided by ultrasound with elastic fusion of MRI images, localizing the ROI when it was radiologically visible. The urologist performed the prostate contour on the ultrasound image before the ablation. At the end of the HIFU a contrast-enhanced ultrasound (CEUS) was performed with intravenous Injection of sulfur hexafluoride (Sonovue®) microbubbles to check the effectiveness of the treatment on the ROI.

## US guided partial ablation

US guidance HIFU was performed using Ablatherm® device (EDAP TMS, Vaulx-en-Velin, France) using a 3MHz transducer for the treatment combined with a 7.5MHz image transducer. Continuous automatic monitoring and re-planning was not performed using the Ablatherm® device.

## Follow-up

**Figure 1:**
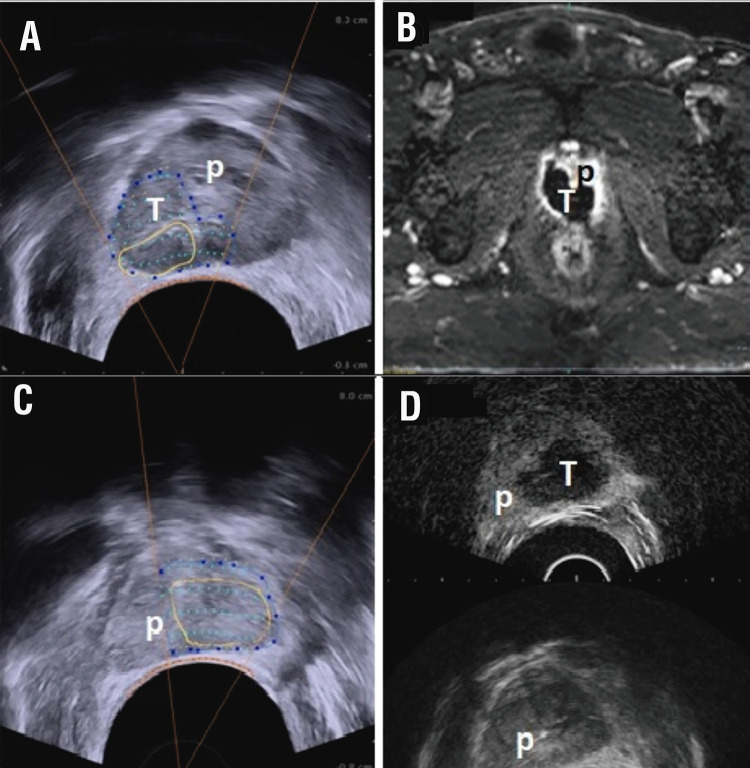
Contrast-enhanced ultrasound image after HIFU. A) Image fusion and treatment planning; B) Treated area control; p=prostate; T=treated area. C-D) (different patient from figures A-B). mpMRI image 1 month after HIFU (hemiablation) + TURP. Patient 77y, PSA: 7.5ng/mL; PCa Gleason 3+4; mpMRI: 10mm lesion 10p PIRADS 5/5, prostate volume 30mL; control PSA: 1.71ng/mL; p=prostate; T=treated area.

Follow-up visits on the 1^st^, 3^rd^, 6^th^, and 12th month post-treatment consisted of a physical examination, and completing the IPSS, ICS, IIEF-5 questionnaires. Patients having no involuntary urine leak and being completely pad free, were defined as continent. The oncological follow-up included PSA measurement on each visit, and the transrectal protocol biopsy (12 core, bi-sextant) with a mpMRI at 1 year of treatment ( Figure 1A-B ). Prior to the 12^th^ month of follow-up patients were not routinely biopsied if not having any clinical suspicion of recurrence. Treatment failure was defined as a positive biopsy in the treated area.

## Statistical Analysis

The Mann-Whitney and chi-square tests were used to compare the statistical significance of differences in medians and proportions, respectively. Fine and Gray multivariable competing risk analyses tested the impact of surgical technique and survival outcomes. Statistical significance was considered at p <0.05. Statistical analyses were performed using SPSS v.22.0 (IBM Corp., Armonk, NY, USA).

## RESULTS

We performed US- and MRI-US guided HIFU PGA in 88 and 52 patients respectively ( Table 1 ). No major baseline differences were present amongst the two cohorts excepts for a higher percentage of Gleason Score 3+4 in the MRI-US guided PGA group (p=0.03). No patients had pre-operative incontinence.

**Table 1:** Comparison of MRI-US guided and the historical series of US guided hemi-ablation (HA) of prostate cancer. Baseline features, Intra-operative data for MRI-US fusion HIFU, Comparison of complications and outcomes.

Variable	US-HIFU	mpMRU-US HIFU	p - value
Number of patients	88	52	
Mean age (SD) years	68 (6.9)	67.6 (7.3)	0.8
Mean BMI (SD)	23.9 (5.9)	25.1 (5.8)	0.8
Mean PSA (SD) ng/mL	7.1 (2.9)	7.7 (2.6)	0.2
Mean Prostate volume (SD) cc	37.2 (12.2)	40.3 (11.6)	0.8
Mean percent of positive cores (SD)	12.4 (10.5)	19.4 (15.2)	0.2
Mean percent of positive core length (SD)	4.9 (5.1)	8.6 (10.6)	0.01
**Gleason Score (%)**			**0.03**
6 (3+3)	73 (83)	36 (68)	
7 (3+4)	15 (17)	16 (32)	
Mean pre-op IPSS score (SD)	5.8 (4.8)	3.6 (3.8)	0.2
Mean pre-op IIEF score (SD)	17.8 (6.7)	22.6 (3.5)	0.1
Number of incontinent patient (%)	0	0	
**mpMRI Informations**			
Prostate mpMRI volume (Median, IQR) cc	-	36.7 (31-48)	
Target Size (MEDIAN, IQR) cc	-	6.3 (4.5 -8.5)	
Number of Targets (MEDIAN, IQR)	-	1 (1 – 2)	
Firing Duration (MEDIAN, IQR) min	-	7.1 (5.2 – 9.7)	
Distance from apex to lower border of target (Median) mm	-	8.6	
Distance from lower to upper border of target (Median) mm	-	23.8	
**Post-treatment results**			
Mean hospitalization time (SD) days	3.1 (0.9)	1.7 (1.1)	0.1
Mean catheterization time (SD) days	2.9 (0.3)	2.4 (0.8)	0.5
**Follow - up at 3 months**			
Mean drop in PSA (SD) ng/mL)	2.5 (3.3)	2.5 (3.4)	0.7
Mean increase in IPSS (SD)	2.4 (4.5)	2.2 (3.7)	0.8
Number of patients with urine leak (%)	14 (15.9)	2 (5.1)	**0.04**
Mean drop in IIEF (SD)	4.2 (6.2)	2 (4.2)	**0.07**
**Complications (%)**	**12 (13.6)**	**3 (6.8)**	**0.2**
Complications requiring hospitalization (%) Clavien >1	7 ( 8 )	1( 2 )	
Complications requiring intervention (%) Clavien 3	2 (2.3)	1( 2 )	

In the mpMRI-US group median mpMRI target volume ablated was 6.3mm3 and the median ablation duration was 7.1min.

No significant differences between the two groups were present in terms of hospital stay (p=0.1) and catheterization time (p=0.5), being 1.7 and 2.4 days respectively for the mpMRI-US PGA group. No statistically significant differences were present in terms of complications (p=0.2) although overall complications were experienced by 6.8% of those undergoing mpMRI-US PGA versus 13.2% in the US PGA group.

At 3 months following the procedures, there was a median increase on IPSS score of 3 (IQR: 1-4); 3 (2.5%) patients demonstrated some urine leak; median drop of IIEF score was 3 (IQR: 1-5). Patients treated with mpMRI-US guided HIFU had significantly lower urine leak (5.1% vs. 15.9%, p=0.04) and a lower drop in IIEF scores (2 vs. 4.2, p=0.07).

Amongst mpMRI-US fusion HIFU, at a median follow-up of 8 months (IQR: 3-18), 25 patients underwent a control biopsy and 6 (26%) had residual cancer in the treated lobe.

## DISCUSSION

In the present study we reported the outcomes of focal therapy being performed with or without mpMRI guidance for the treatment of localized low- to intermediate-risk PCa. To our knowledge, no other studies specifically addressed the impact of mpMRI guidance implementation and of new technologies compared to previously available devices for HIFU focal therapy.

PGA is a method of PCa treatment that involves three main pillars of strategy: cancer localization, accurate delivery of energy to the target and post treatment surveillance ( 14 ).

First, to maximize treatment success, appropriate selection of patients to be treated with PGA is crucial. Several factors should be considered prior to treatment. mpMRI has emerged as a key tool to localize cancer ( 15 - 17 ). In this manuscript we present our series of patients treated with PGA. We also performed systematic biopsies in addition to fusion biopsy to rule out cancer in the other areas of the prostate. In patients with no demonstrable abnormality and TRUS biopsy findings of small volume unifocal cancer, we performed a trans-perineal template guided mapping biopsy to rule out significant multifocal cancers ( 18 ).

Second, mpMRI-US fusion guided HIFU technology essentially addresses the second pillar of the PGA strategy. The current device enables to import MRI images with the ROI marked directly from the institutional image viewer or through an external memory device. The imported images are fused with the TRUS images of the prostate that are acquired during the HIFU planning. The fusion process provides visual guidance of the location of the cancer by fusing mpMRI and TRUS images. The key part in this process is when the treating urologist defines the contour of the prostate in the TRUS images. This process is subjective and involves a learning curve. Gross discrepancies in the prostate contour between the urologist-defined TRUS images and the MRI can lead to fusion failure and inaccurate localization of the cancer. An active collaboration with a dedicated radiologist to mark the lesion with the safety margin of 7-9mm is of paramount importance ( 19 ). This marked area represents the ‘region of interest’ and actually represents the area to be treated. Communication between the treating physician and the radiologist is the key in marking the ROI and in identifying crucial parenchymal landmarks like cysts and calcifications that can eventually help in the confirmation of fusion before treatment.

Recently, Stabile and colleagues detailed results of 1.032 men treated with focal HIFU from 2005 to 2017 and found reduced 5-year re-treatment rate over time. Improvements in patient selection, increasing inclusion of mpMRI visible lesions to select treatment margins and operator learning curves have been suggested as possible reasons for increase in oncological control. However, the impact of these factors on functional outcomes and complications was not assessed ( 20 ).

In the present series, results are not mature enough to prove oncological benefits of an mpMRI guided compared to an ultrasound based focal ablation. Nonetheless, the mpMRI-US guided group yielded significantly improved continence and erectile function compared to the US-guided HIFU series. Although not being statistically significant, a trend towards lower complications was also observed. Hence, the possibility of defining cancer location and accurately planning treatment margins may not only play a role in terms of PCa control as suggested by others ( 20 ), but may also provide advantages in terms of functional recovery and complications.

Indeed, despite being rare, complications do still occur with those having obstructive lower urinary tract symptoms yielding increased risk of acute urinary retention. Despite performing TURP in those with >40g prostates and IPSS>7, three men in the mpMRI-US guided HIFU had urinary retention. In this sense, we will soon perform a feasibility study to detail possible benefits of short course androgen deprivation therapy in terms of oncological but also functional results and complications as prostate volume reduction may reduce obstruction-related morbidity ( 21 ).

Third, Contrast Enhanced UltraSound (CEUS) - sulfur hexafluoride (Sonovue®) was used to verify the macroscopic effectiveness of the ablation at the end of the procedure. CEUS was used for the characterization of abdominal tumors (hepatocellular carcinoma, renal cell carcinoma, etc.), assessment of perfusion of different organs, and for the control after ablative of different malignancies ( 22 , 23 ). The use of this promising tool has been demonstrated to distinguish avascularized tissue and viable tissue post-HIFU ( Figure 1C-D ). In our study, this radiological evidence was performed in 28 cases being very useful for verification of the treated area. The correlation between these avascularized areas and treatment effectiveness has not been demonstrated yet. Nonetheless, immediate post-HIFU CEUS potentially allows early implementation of the third focal therapy pillar, namely post-treatment surveillance. The objective of this procedure is to verify whether the zone of avascularized corresponds to the intended zone of treatment, eventually repeating an immediate second HIFU course to target non-completely ablated zones included in treatment planning. Previously, we used early post-operative mpMRI to verify ablation success. Since the introduction of CEUS, early post-operative mpMRI is no longer performed.

Despite not being a study outcome due to insufficient follow-up, we reported mpMRI-US fusion oncological results for the purpose of completeness. Amongst those undergoing the 12 months control biopsy one on four had residual cancer in the treated lobe, in line with the existing PGA literature ( 1 , 12 ). It is important to note that our institution has vast experience in using TRUS guided HIFU device (HIFU) and transitioning to this new technology was smooth. There is a learning curve for using this new technology and experienced urologists and technicians are important as proctors for the initial few cases.

We are faithful believers in the three pillars proposed by Lindner et al., for the focal treatment of PCa ( 24 ). As for the first pillar, i.e. Planning: mpMRI to identify the ROI and prostate biopsies by TPMB or mpMRI targeted techniques are essential tests to be performed for the identification and location of PCa. As for the second pillar, i.e. Treatment: mpMRI-US guided HIFU to enhance treatment precision and exclusion of mpMRI anteriorly located lesions, as HIFU- induced coagulative necrosis occurs at a focal length of 40mm ( 10 ). As for the third pillar, i.e. Control: CEUS allows checking the area of cavitation and avascularization produced, acquiescing possible extensions of treatment during the same surgical procedure. MRI-US fusion represents a significant advancement in the second pillar of PGA and HIFU application for prostate cancer and potentially also in the third pillar, as an early post-treatment check allows immediate correction of potential undertreatment.

Finally, although not all patients underwent control biopsies as they did not reach one year follow-up, one on four men had residual PCa at 12 months. These results, despite being in line with recent series, remains a sub-optimal and should be highlighted when discussing focal therapy as a potential treatment option with potential candidates ( 5 ).

Our study has some limitations. First, the improvements described for the mpMR-US guided HIFU group are likely to be related not only to the possibility of mpMRI-image fusion, but also likely to reflect technological improvements of a new device (Focal One®) compared to an older one (Ablatherm®). Second, despite surgeons already having a large focal HIFU ablation experience before the study initiation, the focal therapy learning curve has never been specifically assessed and its assessment may also contribute to outcomes amelioration. Third, lesion and treated volumes, which are important variables in focal therapy, were not available for the US guided ablation, thus hampering the comparison with mpMRI-US guided HIFU.

## CONCLUSIONS

HIFU treatment guided by MRI-US fusion images may allow improved functional outcomes and complications compared to US guided HIFU alone. Further analysis should be performed to confirm benefits of mpMRI implementation in HIFU partial gland ablation, both in treatment planning and delivery, and to investigate its potential oncological benefits.
